# An Exploratory Study into Objective and Reported Characteristics of Neuropathic Pain in Women with Chronic Pelvic Pain

**DOI:** 10.1371/journal.pone.0151950

**Published:** 2016-04-05

**Authors:** Lucy H. R. Whitaker, Jen Reid, Alex Choa, Stuart McFee, Marta Seretny, John Wilson, Rob A. Elton, Katy Vincent, Andrew W. Horne

**Affiliations:** 1 MRC Centre for Reproductive Health, University of Edinburgh, The Queen’s Medical Research Institute, 47 Little France Crescent, Edinburgh, EH16 4TJ, United Kingdom; 2 The University of Edinburgh Medical School, Chancellor’s Building, 49 Little France Crescent, Edinburgh EH16 4SB, United Kingdom; 3 Department of Anaesthesia, Royal Infirmary of Edinburgh, 51 Little France Crescent, Edinburgh, EH16 4SA, United Kingdom; 4 Centre for Population Health Sciences, University of Edinburgh, Medical School, Teviot Place, Edinburgh EH8 9AG, United Kingdom; 5 Nuffield Department of Obstetrics and Gynaecology, University of Oxford, John Radcliffe Hospital, Oxford, OX3 9DU, United Kingdom; Boston Children’s Hospital and Harvard Medical School, UNITED STATES

## Abstract

Chronic pelvic pain (CPP) affects 5.7–26.6% women worldwide. 55% have no obvious pathology and 40% have associated endometriosis. Neuropathic pain (NeP) is pain arising as a consequence of a lesion/disease affecting the somatosensory system. The prevalence of NeP in women with CPP is not known. The diagnosis of NeP is challenging because there is no gold-standard assessment. Questionnaires have been used in the clinical setting to diagnose NeP in other chronic pain conditions and quantitative sensory testing (QST) has been used in a research setting to identify abnormal sensory function. We aimed to determine if women with chronic pelvic pain (CPP) have a neuropathic pain (NeP) component to their painful symptoms and how this is best assessed. We performed an exploratory prospective cohort study of 72 pre-menopausal women with a diagnosis of CPP. They underwent a clinician completed questionnaire (DN4) and completed the S-LANSS and PainDETECT^™^ questionnaires. Additionally QST testing was performed by a clinician. They also completed a patient acceptability questionnaire. Clinical features of NeP were identified by both questionnaires and QST. Of the women who were NeP positive, 56%, 35% and 26% were identified by the S-LANSS, DN4 and PainDETECT^™^ respectively. When NeP was identified by questionnaire, the associated laparoscopy findings were similar irrespective of which questionnaire was used. No subject had entirely unchanged QST parameters. There were distinct loss and gain subgroups, as well as mixed alteration in function, but this was not necessarily clinically significant in all patients. 80% of patients were confident that questionnaires could diagnose NeP, and 90% found them easy to complete. Early identification of NeP in women with CPP with a simple questionnaire could facilitate targeted therapy with neuromodulators, which are cheap, readily available, and have good safety profiles. This approach could prevent unnecessary or fertility-compromising surgery and prolonged treatment with hormones.

## Introduction

Chronic pelvic pain (CPP) has a major impact on women of reproductive age. It has a prevalence ranging from 5.6% to 26.6% of the female population worldwide [1]. It has a significant influence on quality of life, and represents a considerable socioeconomic burden [2, 3]. CPP encompasses a variety of symptoms including dysmenorrhoea, dyspareunia, dyschezia and dysuria, as well as pelvic visceral or muscle pain.

Painful pelvic symptoms can be associated with specific gynaecological conditions, such as endometriosis or adenomyosis, affecting approximately 40% of women with CPP. They are also associated with non-gynaecological conditions such as irritable bowel syndrome (IBS), interstitial cystitis or musculoskeletal problems. However, up to 55% of women with CPP have no obvious underlying pathology [4]. Neuropathic pain (pain caused by a lesion or disease of the somatosensory nervous system; NeP) can exist in both women with endometriosis and women in whom there is no macroscopic cause for pain seen, either on imaging or at laparoscopy [5]. Medical treatment for NeP using neuromodulators (antidepressants and anticonvulsants) is cheap, easily available and well tolerated [6].

The molecular and cellular explanations for the development of NeP in the setting of endometriosis are increasingly robust. Direct nerve invasion by endometriosis lesions [7] and neuroangiogenesis [8,9] can contribute to peripheral sensitisation and the development of nociceptive memory [10]. In women without obvious organic pathology, it is postulated that a prior inflammatory insult can stimulate a similar cascade of changes [11]. In women with and without obvious visible pelvic pathology, amplification within the central nervous system secondary to a variety of external factors (e.g. sleep deprivation, endocrine dysfunction and psychological distress) may also contribute to their experience of pain [12]. Equally the central model may explain the increased prevalence of other clinical entities such as regional pain syndromes, fibromyalgia and chronic fatigue syndrome.

Unfortunately, the diagnosis of NeP can be challenging. There is no recognised gold standard for assessment. Some studies have used either quantitative sensory testing (QST) or expert opinion as their gold standard [13] [14]. QST aims to objectively assess peripheral nerve function using calibrated instruments to determine thresholds of sensation including temperature, punctate and vibration stimuli. Both are impracticable in routine clinical practice—QST is too time consuming and waiting times for pain medicine specialists are long [15]. A surrogate is NeP questionnaires. PainDETECT^™^[16] is commonly used in clinical practice and has been well validated in NeP outwith the pelvis. It is patient completed; asks them to localise their pain, indicate fluctuations in timing and then scores the extent to which known neuropathic features contribute. Other NeP questionnaires include the DN4 [17] and the S-LANSS [13]. The DN4 is clinician completed and includes clinical examination for presence of allodynia (the sensation of pain in response to a non-noxious stimulus) and hypoesthesia. The S-LANSS is patient completed and has binary answers to the presence or absence of the features of neuropathic pain. As with PainDETECT^™^, neither of these has been validated within the pelvis. All three are used extensively in chronic pain management outside of the reproductive tract.

Some researchers have suggested subgrouping within women with NeP as an adage to personalising therapy [18]. When PainDETECT^™^ is used, five subgroups have been identified in patients with NeP with other underlying clinical pathology The subgroups identified by principle component analysis are defined on the basis of the pattern of loss and/or gain of function and are thus best represented pictorially (see [19] for a full description of these subtypes). These may represent different underlying mechanisms generating neuropathic pain and thus potentially may have utility in guiding choice of adjunctive analgesia. Clear subgroups have been found in those with post-herpetic neuralgia and diabetic neuropathy. This has not been investigated in women with CPP.

QST assesses the response of both un-myelinated C and myelinated A fibres, and with the use of calibrated instruments to determine sensation threshold to a variety of stimuli, aims to provide reproducible results (Table 1). The viscerosomatic convergence within the spinal column is reflected in cutaneous sensory changes in those with visceral hypersensitivity. This has been well documented, including within the female pelvis [20]. The majority of the body of work has been done by the German Research Network on Neuropathic Pain (DNFS) who have established protocols for research and databases for thresholds. QST has previously been used as a gold standard in other trials for diagnosis of NeP, and has also been used specifically in CPP of varying aetiologies [21].

**Table 1:** Summary of information related to assessment of different peripheral and central somatosensory channels.

Type of stimulus	Peripheral sensory fibre	Central pathway	Bedside examination	QST
**Thermal**				
Cold	Aδ	Spinothalamic	Cold reflex hammer, cold thermorollers	Computer controlled thermal testing device
Warm	C	Spinothalamic	Warm thermorollers	Computer controlled thermal testing device
Heat pain	C, Aδ	Spinothalamic	Warm/hot thermorollers	Computer controlled thermal testing device
**Mechanical**				
Static light touch	Aβ	Lemniscal	Q-tip	Calibrated von Frey hairs
Vibration	Aβ	Lemniscal	Tuning fork	Vibrameter
Brushing	Aβ	Lemniscal	Brush/cotton swab	Brush
Pinprick	Aδ, C	Spinothalamic	Pin	Calibrated pins
Blunt pressure	Aδ, C	Spinothalamic	Examiner’s thumb	Alogmeter

We hypothesised that within a tertiary hospital gynaecology service there would be patients with chronic pelvic pain who had NeP features to their pain and that NeP pain questionnaires could be used to ascertain this.

We carried out an exploratory clinical study to assess

- Whether women with CPP of varying aetiology had features suggestive of NeP on questionnaire
- How well the NeP questionnaires correlated with each other
- Whether rates of NeP were affected by underlying aetiology
- Whether there was an objective alteration in sensory symptoms in women with CPP
- Whether questionnaires are acceptable to patients for identifying NeP

## Materials and Methods

### Study setting

The study was undertaken in the Gynaecology Outpatient Department of the Simpson Centre for Reproductive Health (SCRH) in the Royal Infirmary of Edinburgh, UK. The study was conducted from November 2012 –March 2014.

### Ethical approval

Ethical approval was obtained from the Scotland A Research Ethics Committee (REC No: 12/SS/0149). All patients provided written consent.

### Study participants

Patients attending the Edinburgh Endometriosis Centre of Excellence (www.exppectedinburgh.co.uk), the NHS Lothian Pelvic Pain Service and General Gynaecology clinics were invited to take part, and all participants provided informed consent. Inclusion criteria were as follows: women aged 18 to 55 years; CPP defined as ‘intermittent or constant pain in the lower abdomen or pelvis of at least six months duration, not occurring exclusively with menstruation or intercourse and not associated with pregnancy’ [22]. Patients were excluded if the pain was not localized to the deep pelvis region, such as women with vulvodynia.

#### Sample size

A previous six-month retrospective audit of patients with CPP from a tertiary clinic suggested a 70% negative diagnosis for the presence of NeP (unpublished data). Based on this audit, using the properties of the binomial distribution in the sample of true negatives the sample size for this clinical study was calculated to be 72 [23] and was powered for specificity of 85% with confidence limits of +/-10%, based on validation of NeP questionnaires outside the reproductive tract.

#### Questionnaires

We used the S-LANSS [13], DN4 [17] and PainDETECT^™^ [16], with the original wording and layout but with a local publishing format. We designed an acceptability questionnaire that was patient completed.

#### Quantitative Sensory Testing

**Fig 1:**
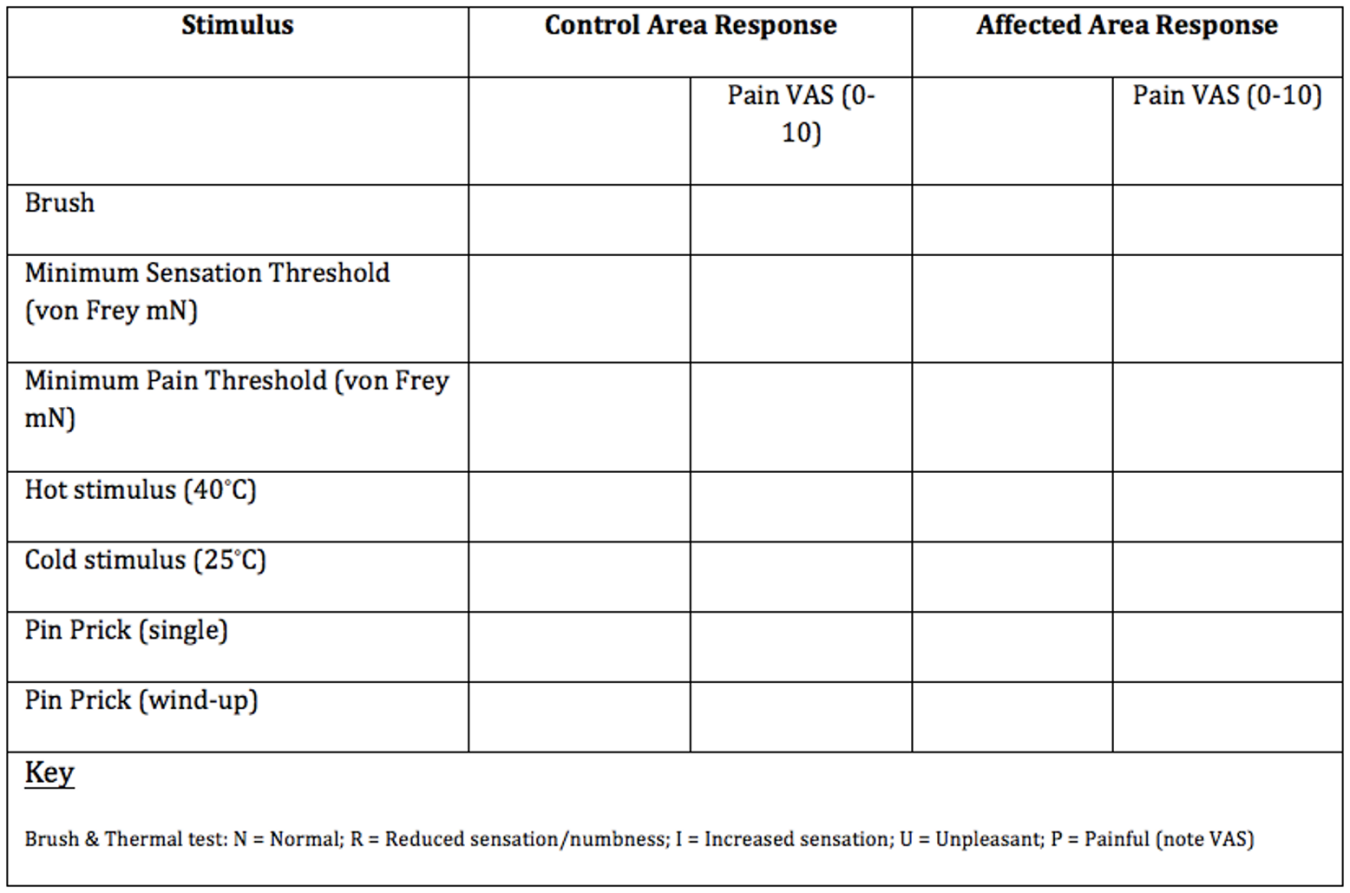
Lothian Chronic Pain service Modified QST protocol.

Modified QST testing was undertaken by a clinician using the NHS Lothian Chronic Pain Services modified QST Protocol (Fig 1). The area of maximal change on the abdomen was identified using a sense brush (Somedic, Sweden 10-620-0001). If no abnormality was detected the area that pain was localised to was used for the test area. The control area was just below the xiphisternum. With the subjects’ eyes shut, von Frey filaments (Stoelting, USA 58011) were used to calculate minimum sensory and pain thresholds. For sensory threshold a detection of three out of five touches was deemed threshold. For pain threshold, a single touch only was used. Roll temp (calibrated metal rollers set to 25 and 40°C; Somedic, Sweden 10-610-0001) were used to elicit response to temperature stimulus relative to the control site (unchanged, decreased, increased, unpleasant or painful. Finally single pinprick and wind-up (five rapid touches) response were elicited using a sterile neurotip. If at any point the stimulus was considered painful it was rated on a visual analogue scale (VAS). ‘Loss or gain of function’ (i.e. increased/decreased sensitivity in the affected area was relative to the control site at the xiphisternum.

#### Demographic data collection

Basic demographics were collected, as well as menstrual phase during assessment, current hormonal treatment, analgesics and neuromodulator use. Following QST, the subjects’ clinical notes were reviewed for their attending clinician’s impression of aetiology, and findings at laparoscopy. If the participant had previously been reviewed by a pain medicine specialist, their diagnosis was also documented.

#### Data Analysis

Descriptive statistics were used to analyse baseline characteristics. We used a χ^2^ test to compare dichotomous data. Statistical analysis was performed using GraphPad PRISM Version 6. All women had their QST results sub-analysed for grouping into either pure loss, or gain of function, or mixed neuropathic disorder compared to control area. They also had their PainDETECT^™^ parameters individually divided to assess if there were similar subgrouping seen to other neuropathic disorders [19]. This includes 5 subgroups based on pattern of deviation from the mean of the individual parameters (burning, prickling, mechanical allodynia, painful attacks, thermal allodynia, numbness and pressure evoked pain). These subgroups have previously been determined using hierarchical cluster analysis on 4200 patients completing PainDETECT^™^ (19). Medication was not controlled for in the analysis due to the small number of patients.

## Results

### Participant characteristics

The average age of the women who agreed to take part was 34 (range 20–54). Thirty-eight women (53%) were nulliparous and, of those who were parous, 38% had undergone caesarean section. Forty-two women (58%) were taking a hormonal preparation and 23 (32%) were taking a neuromodulator. All participants with the exception of two had undergone laparoscopy and all those with a diagnosis of endometriosis had their staging noted with the exception of two, who did not have an operation note available. The average duration of symptoms was eight years and nearly half of the women had had two or more operations for investigation and treatment of their pain (Table 2)

**Table 2:** Characteristics of subjects.

Age, mean (SD) [range]	34.5 (8.85) [20–54]
Parity n (percentage)	
- Nulliparous	38 (52.8)
- Parous	34 (47.2)
- Previous CS	13 (18.1)
Hormone treatment n (percentage)	
- Nil	30 (41.7)
- Combined Oral Contraceptive Pill	10 (13.9)
- Progesterone Only Pill	2 (2.8)
- DepoProvera^™^	2 (2.8)
- Levonogesterol releasing intrauterine system	11 (15.3)
- GnRH analogue	2 (2.8)
- Oophorectomy with add back Hormone replacement therapy	2 (2.8)
Laparoscopic findings n (percentage)	
- No visible pathology	26 (37.1)
- Endometriosis	32 (45.7)
Stage I	12 (37.5)
Stage II	7 (21.9)
Stage III	3 (9.4)
Stage IV	8 (25.0)
Stage not documented	2
- Adhesions	7 (10.0)
- Dermoid cysts	5 (7.1)
Total number of operations: mean (SD) [range]	1.9 (1.1) [0–5]
Pain medication n (percentage)	
- Analgesics (8 not documented)	57 (90.5)
- Neuroleptics	23 (32.9)
Global pain score /100: mean (SD) [range]	31.9 (26.3) [0–87]
Duration of symptoms in months: mean (SD) [Range]	92 (94) [6–456]

### Summary of questionnaire findings

**Fig 2:**
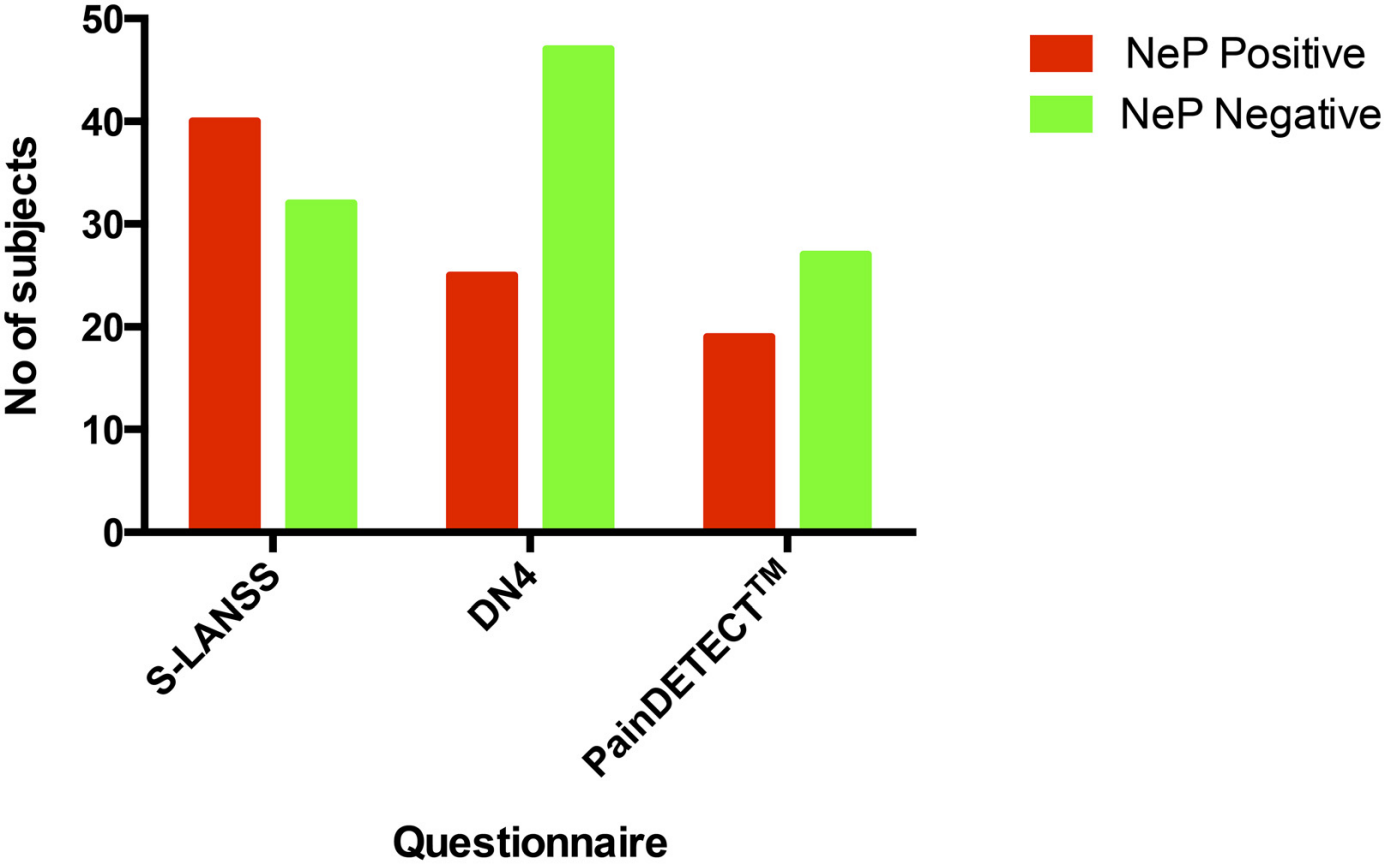
Questionnaire outcomes.

**Fig 3:**
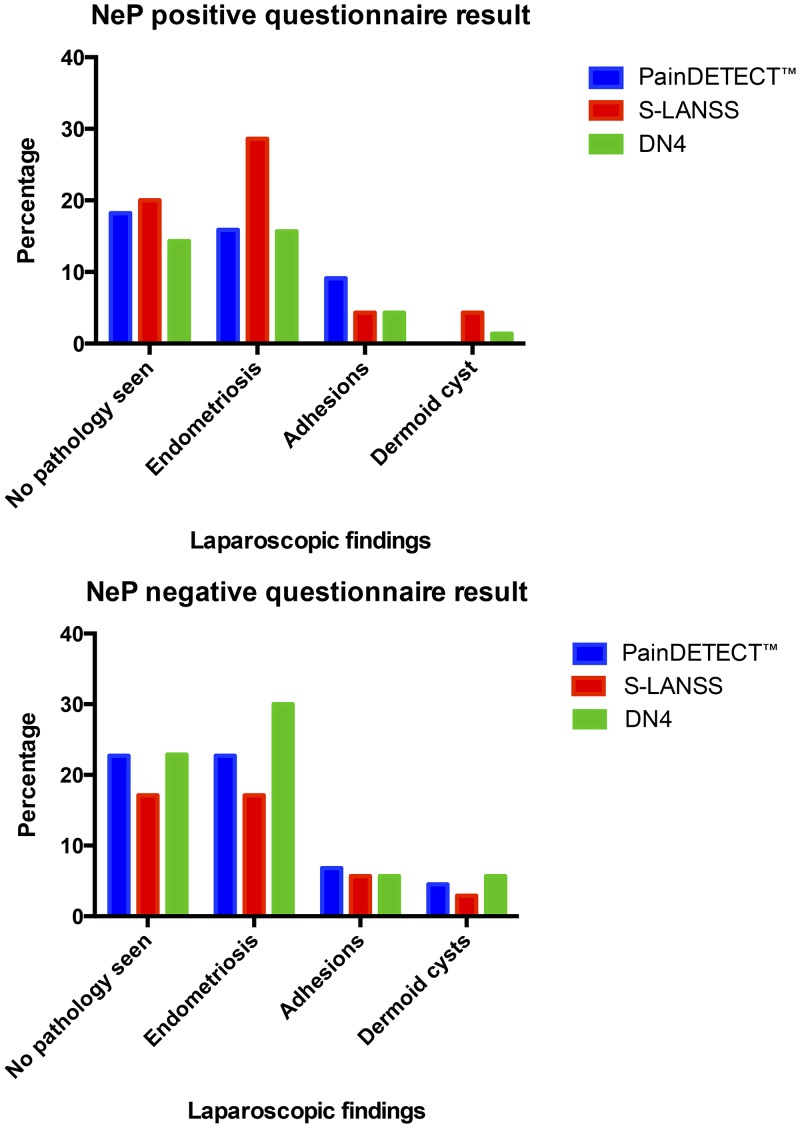
Laparoscopic findings in women with questionnaires positive and negative for NeP.

Participants with NeP were identified by all three questionnaires (Fig 2), with positive rates of 56%, 35% and 26% by the S-LANSS, DN4 and PainDETECT^™^, respectively. The overall agreement between questionnaires was 41.7%. Agreement was higher for a negative, rather than a positive diagnosis (25.0% and 16.6% respectively). Internal consistencies in this sample, as measured by Cronbach’s alpha, were 0.72 for PainDETECT^™^, 0.61 for S-LANSS and 0.67 for DN4. Twelve of the participants had been seen by a pain medicine specialist, of whom nine had a clinical diagnosis corresponding to the questionnaire diagnosis. The correlation with clinical diagnosis varied between questionnaires (Table 3). The rates of NeP with each questionnaire were compared to the findings seen at laparoscopy (Fig 3). The incidence of NeP did not differ significantly between the women with a laparoscopic diagnosis of endometriosis and those with no obvious pathology, irrespective of which questionnaire was used (S-LANSS Χ^2^ = 0.67, N = 72, p = 0.51; DN4 Χ^2^ = 0.32, N = 72, p = 0.75 and PainDETECT^™^ Χ^2^ = 0.20, N = 35, p = 0.85). It was not possible to subdivide the 19 participants with PainDETECT^™^ NeP positive into the five distinct phenotypes seen with other underlying aetiologies.

**Table 3:** Correlation between clinical diagnosis and NeP questionnaire outcome.

	S-LANSS	DN4	PainDETECT^™^
Sensitivity	0.8889	0.7778	0.8571
(95% CI)	(0.5175 to 0.9972)	(0.3999 to 0.9719)	(0.4213 to 0.9964)
Specificity	0.3333	1	0.5
(95% CI)	(0.008 to 0.9057)	(0.2924 to 1)	(0.0126 to 0.9874)
Positive Predictive Value	0.8	1	0.8571
(95% CI)	(0.4439 to 0.9748)	(0.5904 to 1)	(0.4213 to 0.9964)
Negative Predictive Value	0.5	0.6	0.5
(95% CI)	(0.0126 to 0.9874)	(0.1466 to 0.9473)	(0.0126 to 0.9874)

### Quantitative sensory testing results

**Fig 4:**
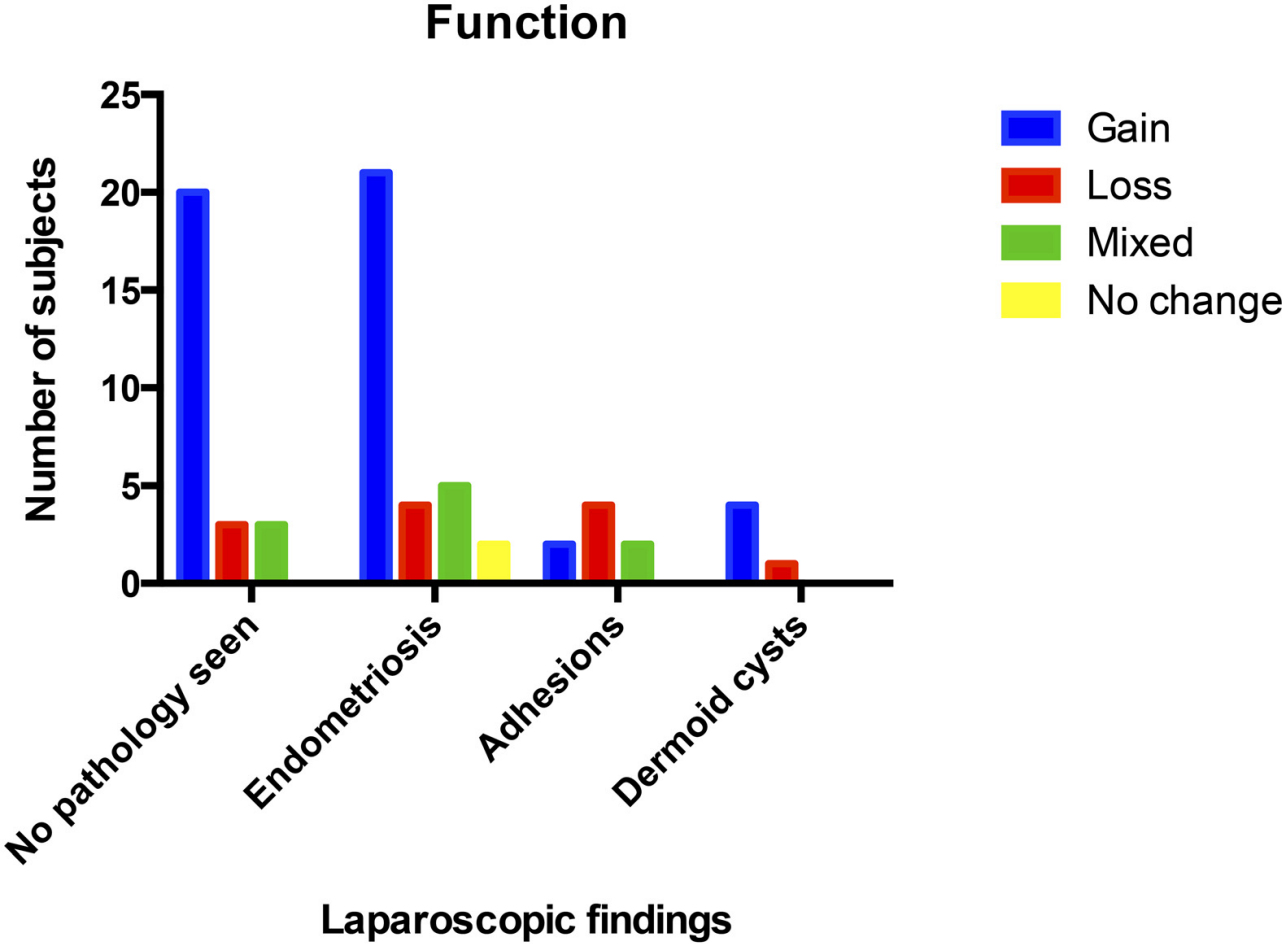
Sensory profiles for punctate pain threshold and thermal stimulus.

No participants had entirely unchanged sensory findings in their ‘pain area’. The degree of sensory alteration varied greatly. When the temperature and punctate pain thresholds were subdivided for loss and gain of function (decreased and increased sensation respectively) there appeared to be no difference in rates of gain of function between endometriosis and those with no obvious pelvic pathology (Fig 4). Only two participants had no marked change in in these two parameters, both of these had Stage I endometriosis.

### Acceptability of questionnaires

Fifty-seven of the subjects (79%) were confident that questionnaires could correctly diagnose NeP in the setting of CPP. This was unaffected by underlying aetiology. Overall the PainDETECT^™^ was favourably viewed by 67 of the subjects (93%) and 65 subjects (90%) felt it was easy to complete. The S-LANSS had similar acceptability levels of 89% and identical ease of completion. The DN4 did not have patient acceptability and ease of completion assessed as this is a clinician-completed form.

## Discussion

Our exploratory clinical study suggests clinical features of NeP are present in over 50% of women with CPP. The true prevalence needs to be ascertained in a larger cohort of women but is likely to be difficult to determine due to the lack of a clinically practical and a robust gold standard for diagnosing NeP. The comparison of questionnaires with a pain medicine specialist’s clinical assessment suggest a high level of sensitivity and positive predictive value but as only a small numbers of patients had had a pain medicine specialist review this cannot be taken as a validation. The rates of endometriosis were not statistically different between the NeP positive and NeP negative groups of women, highlighting the importance of considering co-existing sensory dysfunction with pelvic pain of any underlying aetiology. Sensory alteration was demonstrated in almost all women in the study, but a clinically significant threshold has yet to be established. Questionnaires have the confidence of women and are easily completed.

In addition to the small numbers having independent verification of diagnosis by a pain specialist there are a number of further limitations. The study population was heterogeneous in nature including parity, rates of previous surgery and concomitant medication (both hormonal, neuromodulators and analgesics). The study population was heterogeneous in nature including parity, rates of previous surgery and concomitant medication (both hormonal, neuromodulators and analgesics). However the majority of patients presenting to secondary and tertiary care have either had empirical treatment in the community, are referred from general gynaecology or are representing with ongoing pain, as such whilst the heterogeneity may have confounded results, it is typical of patients presenting to specialist gynaecological care.

Further limitations include the QST protocol used. Visceral hypersensitivity is most accurately determined by rectal hyperreflexia, but this is invasive and often unacceptable to patients. As such, alternative methods of objectively determining sensory abnormalities need to be used. The original DNFS protocols have had modified versions produced in order to facilitate bedside testing, particularly the substitution of the computer controlled thermodes for calibrated thermorollers and the removal of the algometer and vibrameter but they remain time consuming. As such a modified QST protocol was used as the standard to assess NeP in the pelvis. Furthermore, the majority of patients within this study both had childcare commitments and fulltime employment and so a shortened protocol was more acceptable and logistically feasible but does not permit validation of the questionnaires against the DNFS data.

Patient questionnaires are a potential surrogate for specialist clinical assessment. This study underscores their ease of completion and patient confidence in their use. Furthermore, if validated they could potentially be used with confidence in a primary care setting in order to facilitate early identification of NeP and thereby expedite appropriate treatment with a neuromodulator at an early stage of patient presentation.

Timely identification of NeP may also help to reduce the iatrogenic harm of unnecessary surgery and inappropriate medication. In addition, it could reduce the need for many women to be seen in specialist services and allow the initiation of successful treatment in primary and secondary care. This is particularly relevant given the long duration of symptoms in this patient group with their impact on healthcare needs, work productivity, relationships and sexual function. However the degree of variation between the questionnaires in this cohort mean the ideal questionnaire for diagnosis of NeP in CPP has yet to be determined.

The numbers are too small to ascertain whether stage of endometriosis has an impact on the rates of NeP. A larger study is pressingly needed, particularly as upcoming imaging studies such as MEDAL [24] investigating the utility of MRI as a surrogate for laparoscopy may change the way CPP is investigated and managed within secondary and tertiary care. If higher rates of NeP are found in lower stages of disease, this may explain, at least in part, the disconnect between macroscopic finding and severity of pain.

Within this small study, the QST parameters suggest that all three separate function groups exist within the CPP groups. The apparent relationship between loss of function and underlying adhesions is of clinical interest as there is a lack of robust clinical evidence for repeated surgery to divide adhesions [25]. Targeted use of neuromodulators, particularly within this patient group, may improve symptoms and reduce the amount of technically challenging (and often unsuccessful surgery). A substantially larger study is required to determine if subtyping of NeP is possible using the PainDETECT^™^ questionnaire. This has clinical application because the response to neuromodulator therapy can be varied and this maybe due to subsets of altered function within the setting of NeP. Furthermore, it could help within clinical trials to truly determine efficacy as well as facilitating personalised, targeted drug treatment.

In summary, NeP is a major component of CPP irrespective of associated pelvic pathology. This can be demonstrated both using objective clinical assessment and NeP patient questionnaires. These questionnaires could improve both the timing and accuracy of the diagnosis of NeP and are acceptable to women with CPP. They may also facilitate studies into determining the true incidence of NeP in women with CPP and enable timely targeted therapy with neuromodulators.
